# Maternal and neonatal services in Ethiopia: measuring and improving quality

**DOI:** 10.2471/BLT.16.178806

**Published:** 2017-04-25

**Authors:** Maureen E Canavan, Marie A Brault, Dawit Tatek, Daniel Burssa, Ayele Teshome, Erika Linnander, Elizabeth H Bradley

**Affiliations:** a Global Health Leadership Institute, Yale University School of Public Health, 2 Church Street South, New Haven, Connecticut, 06519, United States of America.; b Federal Ministry of Health, Addis Ababa, Ethiopia.

## Abstract

**Problem:**

Maternal and neonatal mortality remains high in low- and middle-income countries, with poor quality of intrapartum care as a barrier to further progress.

**Approach:**

We developed and tested a method of measuring the quality of maternal and neonatal care that could be embedded in a larger national performance management initiative. The tool used direct observations and medical record reviews to score quality in nine domains of intrapartum care. We piloted and evaluated the tool in visits to the 18 lead hospitals that have responsibility to promote and coordinate quality improvement efforts within a hospital cluster in Ethiopia. Between baseline and follow-up assessments, staff from a national quality collaborative alliance provided hospital-based training on labour and delivery services.

**Local setting:**

Ethiopia has invested in hospital quality improvement for more than a decade and this tool was integrated into existing quality improvement mechanisms within lead hospitals, with the potential for scale-up to all government hospitals.

**Relevant changes:**

Significant improvements in quality of intrapartum care were detected from baseline (June–July 2015) to follow-up (February–March 2016) in targeted hospitals. The overall mean quality score rose from 65.6 (standard deviation, SD: 10.5) to 91.2 (SD: 12.4) out of 110 items (*P* < 0.001).

**Lessons learnt:**

The method was feasible, requiring a total of 3 days and two to three trained data collectors per hospital visit. It produced data that detected substantial changes made during 8 months of national hospital quality improvement efforts. With additional replication studies, this tool may be useful in other low- and middle-income countries.

## Résumé

**Problème:**

La mortalité maternelle et la mortalité néonatale restent élevées dans les pays à revenu faible et intermédiaire, où la qualité insuffisante des soins pratiqués pendant l'accouchement constitue un frein à la progression dans ce domaine.

**Approche:**

Nous avons conçu et testé une méthode pour évaluer la qualité des soins dispensés aux mères et aux nouveau-nés, qui pourrait être intégrée à une initiative nationale de gestion des performances de plus grande envergure. Grâce à des observations directes et à l'examen des registres médicaux, notre outil a permis d'attribuer un score de qualité dans neuf domaines de soins intrapartum. Nous avons appliqué et testé notre outil dans le cadre de la visite des 18 hôpitaux principaux ayant chacun la responsabilité de promouvoir et de coordonner les efforts d'amélioration de la qualité au sein d'un groupement d'hôpitaux en Éthiopie. Entre les évaluations de départ et les évaluations ultérieures de suivi, le personnel d'une alliance nationale de collaboration pour la qualité a dispensé des formations dans les hôpitaux sur les services réalisés pendant le travail et l'accouchement.

**Environnement local:**

L'Éthiopie investit dans l'amélioration de la qualité de ses hôpitaux depuis plus de dix ans, et cet outil a été intégré aux mécanismes existants d'amélioration de la qualité de ces hôpitaux principaux, avec la perspective de l'étendre éventuellement à tous les hôpitaux gouvernementaux.

**Changements significatifs:**

Des améliorations considérables de la qualité des soins intrapartum ont été constatées entre les données initiales (juin à juillet 2015) et les données de suivi (février à mars 2016) dans les hôpitaux ciblés. Le score global moyen de la qualité est passé de 65,6 (écart type, ET: 10,5) à 91,2 (ET: 12,4) pour les 110 éléments évalués (*P* < 0,05).

**Leçons tirées:**

La méthode était réalisable, nécessitant pour chaque visite trois jours et entre deux et trois collecteurs de données expérimentés. Elle a permis d'obtenir des données reflétant les changements importants survenus en huit mois d'efforts d'amélioration de la qualité des hôpitaux nationaux. Moyennant des études de réitération complémentaires, cet outil pourrait être utile dans d'autres régions à revenu faible et intermédiaire.

## Resumen

**Situación:**

La mortalidad materna y neonatal sigue siendo alta en países con ingresos bajos y medios, siendo la escasa calidad de atención durante el parto un impedimento para hacer progresos.

**Enfoque:**

Se desarrolló y probó un método de cálculo de la calidad de la atención materna y neonatal que podría aplicarse en una gran iniciativa nacional de gestión del rendimiento. La herramienta utilizó observaciones directas y revisiones de historiales médicos para puntuar la calidad en nueve dominios de atención durante el parto. La herramienta se pilotó y evaluó en visitas a los 18 hospitales más importantes con responsabilidad para fomentar y coordinar esfuerzos de mejora de la calidad de un conglomerado de hospitales en Etiopía. Entre las primeras evaluaciones y las de seguimiento, el personal de una colaboración nacional de calidad proporcionó formación hospitalaria sobre el parto.

**Marco regional:**

Etiopía ha invertido en mejoras de la calidad de los hospitales durante más de una década y esta herramienta se ha integrado a los mecanismos existentes de mejora de la calidad en los hospitales más importantes, con potencial para llegar a todos los hospitales públicos.

**Cambios importantes:**

Se detectaron mejoras significativas en la calidad de la atención durante el parto desde el principio (junio-julio de 2015) hasta el seguimiento (febrero-marzo de 2016) en los hospitales estudiados. La calidad media general obtuvo una puntuación de 65,6 (desviación estándar, DE: 10,5) a 91,2 (DE: 12,4) de 110 elementos (*P* < 0,05).

**Lecciones aprendidas:**

El método fue viable, necesitando un total de tres días y de dos a tres recopiladores de datos con formación por visita hospitalaria. Se generaron datos que detectaron grandes cambios durante los ocho meses de trabajos de mejora de la calidad de los hospitales. Con otros estudios de reproducción, esta herramienta podría resultar útil en otros países con ingresos bajos y medios.

## ملخص

**المشكلة:**

لا يزال معدل وفيات الأمهات وحديثي الولادة مرتفعًا في البلدان المنخفضة والمتوسطة الدخل، بالإضافة إلى ضعف الجودة النوعية للرعاية أثناء الولادة والتي تمثل عائقًا أمام إحراز المزيد من التقدم.

**الأسلوب:**

قمنا بتطوير واختبار طريقة لقياس الجودة النوعية لرعاية الأمهات وحديثي الولادة يمكن إدماجها في مبادرة وطنية أكبر لإدارة الأداء **. ** وقد اعتمدت الأداة على الملاحظات المباشرة ومراجعات السجلات الطبية لتسجيل الجودة النوعية في تسعة مجالات للرعاية أثناء الولادة. وقد قمنا بتجربة وتقییم الأداة في زيارات إلی 18 مستشفى رئيسية مسؤولة عن تعزيز وتنسيق جهود تحسین الجودة النوعية داخل مجموعة من المستشفيات في إثيوبيا. وفيما بين التقييمات الأساسية وتقييمات المتابعة، قدم موظفون من جمعية تعاونية وطنية معنية بالجودة النوعية تدريبًا في المستشفيات على خدمات المخاض والولادة.

**المواقع المحلية:**

لقد استثمرت إثيوبيا في تحسين الجودة النوعية للمستشفيات على مدار أكثر من عقد من الزمان، وتم دمج هذه الأداة في آليات تحسين الجودة القائمة داخل المستشفيات الرائدة، مع إمكانية التوسع في جميع المستشفيات الحكومية.

**التغييرات ذات الصلة:**

تم الكشف عن تحسينات كبيرة في الجودة النوعية للرعاية أثناء الولادة من خط الأساس (يونيه/حزيران – يوليو/تموز 2015) إلى المتابعة (فبراير/شباط – مارس/آذار 2016) في المستشفيات المستهدفة. وارتفع المتوسط العام لنتيجة الجودة النوعية من 65.6 (الانحراف المعياري: 10.5) إلى 91.2 (الانحراف المعياري: 12.4) من بين 110 عناصر ( *نسبة الاحتمال *> 0.05).

**الدروس المستفادة:**

أثبتت الطريقة جدواها، حيث تتطلب الأمر 3 أيام في المجمل، وما بين 2 إلى 3 جامعي بيانات مدربين في كل زيارة إلى المستشفى. وأنتجت الطريقة بيانات كشفت عن التغييرات الجوهرية التي أُجريت خلال 8 أشهر من الجهود الوطنية لتحسين الجودة النوعية في المستشفيات. ومع دراسات النسخ المتماثل، قد تكون هذه الأداة مفيدة في البلدان الأخرى ذات الدخل المنخفض والمتوسط.

## 摘要

**问题:**

由于分娩护理质量较差、阻碍进一步发展，导致中低收入国家的孕产妇及新生儿死亡率仍然居高不下。

**方法:**

我们制定并测试了一个衡量孕产妇及新生儿护理质量的方法，该方法可被纳入大型国家绩效管理举措中。该方法采用直接观察与医疗记录审查对九大分娩护理领域的质量进行评分。我们通过拜访 18 家负责在埃塞俄比亚医院集群内部推进并协调质量改进工作的领头医院，试行并评估了该方法。在基础评估至后续评估期间，由国家质量协作组的工作人员提供与产程及分娩服务相关的院内培训。

**当地状况:**

埃塞俄比亚已投资医院质量改进十余年，且此方法已整合入领头医院的现有质量改进机制，并可能推广至所有政府医院。

**相关变化:**

我们发现目标医院在基础评估（2015 年 6-7 月）至后续评估（2016 年 2-3 月）期间的分娩护理质量有显著改进。平均质量总分由 65.6（标准偏差，SD： 10.5）上升至 91.2 (SD： 12.4)，评估项共计 110 项 (*P* < 0.05)。

**经验教训:**

该方法具有可行性，每次医院访问共计需要 3 天及两至三名经过培训的数据采集员。该方法能够提供监测为期 8 个月的国家医院质量改进工作中所取得的重大变化的数据。通过其它重复性研究，此方法在其它中低收入国家可能会有使用价值。****

## Резюме

**Проблема:**

Материнская и неонатальная смертность в странах с низким и средним уровнем доходов остается высокой, а низкое качество интранатального медицинского обслуживания препятствует дальнейшему прогрессу.

**Подход:**

Мы разработали и опробовали метод измерения качества медицинского обслуживания для матерей и новорожденных, который можно было бы включить в более масштабную инициативу по управлению эффективностью деятельности на национальном уровне. В качестве инструмента использовались прямые наблюдения и обзоры медицинской документации для оценки качества в девяти областях интранатального медицинского обслуживания. Мы опробовали и оценили этот инструмент при посещении 18 ведущих больниц, которые отвечают за продвижение и координацию мероприятий по повышению качества медицинского обслуживания в кластере больниц в Эфиопии. В период между исходными и последующими оценками сотрудники национального сотрудничества в области качества предоставляли обучение на базе больниц по вопросам родовспоможения.

**Местные условия:**

Эфиопия в течение более чем десяти лет осуществляла инвестиции с целью повышения качества медицинского обслуживания в больницах, и этот инструмент был интегрирован в существующие механизмы повышения качества обслуживания в ведущих больницах с возможностью расширения масштабов до применения во всех государственных больницах.

**Осуществленные перемены:**

Значительные улучшения качества интранатального медицинского обслуживания были выявлены в целевых больницах при сравнении исходных показателей (июнь-июль 2015 г.) и последующих оценок (февраль-март 2016 г.). Общий средний показатель качества вырос с 65,6 (стандартное отклонение, СО: 10,5) до 91,2 (СО: 12,4) из 110 пунктов (*P* < 0,05).

**Выводы:**

Для применения этого метода требовалось в общей сложности три дня и от двух до трех подготовленных специалистов по сбору данных на одно посещение больницы. С помощью этого метода были получены данные, свидетельствующие о существенных изменениях, произошедших в течение 8 месяцев в ходе мероприятий по повышению качества медицинского обслуживания в больницах. При проведении повторных исследований было продемонстрировано, что этот инструмент может быть полезен в других странах с низким и средним уровнем дохода.****

## Introduction

Maternal mortality remains high in most low-and middle-income countries, and poor quality of intrapartum care limits further progress.^1^^,^^2^ Pregnancy- or childbirth-related complications lead to more than 380 preventable deaths of women per day in these countries.^3^ Researchers have underscored the importance of improving the quality of labour and delivery care for continued reduction in preventable maternal and neonatal deaths.^4^^,^^5^

Reliable and valid evaluation of quality of intrapartum care is paramount for addressing high maternal and neonatal mortality rates in low- and middle-income countries; yet progress has been slow. Recent efforts to develop and validate instruments for use in sub-Saharan Africa are encouraging,^6^^,^^7^ but experience integrating such tools into routine practice is limited. Studies of the quality of maternal and neonatal care in low- and middle-income countries largely focus on evaluating the quality of a single intervention^6^^,^^8^ rather than multiple aspects of intrapartum care, and often in one hospital at a single point in time, thus limiting the generalizability of the findings. Furthermore, few studies have reported on quality measurement efforts embedded in broader national and international quality improvement strategies; this limits their impact globally.

We sought to develop and test a method of measuring the quality of maternal and neonatal care that could be embedded in a larger national performance management initiative. We undertook the study in Ethiopia, where facility-based births are increasing,^9^ and where the country has invested in hospital quality improvement since 2003.^10^^,^^11^ Although we were unable to measure patient outcomes, we examined both processes of care and the capacity of facilities to provide quality-of-care processes, with a focus on elements of intrapartum care.

## Local setting

In 2014, as part of national hospital reform efforts, the Ethiopian Federal Ministry of Health identified the quality of hospital-based labour and delivery care as key priorities for improvement. The medical services directorate of the health ministry convened a committee of ministry staff and international partners to create a tool and a protocol for conducting site visits and collecting data on intrapartum care quality. We piloted and evaluated the tool and assessment process in the 18 hospitals within the Ethiopia Alliance for Hospital Quality.^10^ The alliance is a national quality collaborative initiative, in which the approximately 140 government hospitals are each assigned to a cluster with one lead hospital to work on prioritized hospital quality issues. Lead hospital status is therefore an indicator of generally better performance and special responsibility to promote and coordinate quality improvement efforts within a hospital cluster. In the first 18-month cycle, the priority quality target was adherence to the national guidelines for reforming hospital management.^12^ The second 18-month cycle focused on improving patient satisfaction, and the third cycle focused on improving hospital labour and delivery care.

Components of the hospital performance management framework in Ethiopia have previously been described in detail.^10^^–^^13^ The assessment tool and process of evaluation was integrated into existing mechanisms within the 18 lead hospitals, with the potential for scale-up to all government hospitals. Here, we report the experience of implementing the tool in these hospitals.

## Approach

A longitudinal study was conducted in all the 18 lead hospitals. They represented hospitals from the five most populous regions of Ethiopia. Baseline data from 2015 showed that on average (mean values), these hospitals had 136 inpatient beds (standard deviation, SD: 71 beds), provided 113 937 (SD: 68 431) outpatient visits and 7091 (SD: 4492) inpatient admissions annually, and had 2893 (SD: 1835) deliveries. Hospitals were staffed by an average of 24 (SD: 11) physicians (including 3 surgeons) and 131 (SD: 42) nurses.

We developed the assessment tool (available from the corresponding author) from the Ethiopian *National maternal and newborn care service guidelines* (Burssa D, Ethiopian Ministry of Health, unpublished data, February 2015) and the World Health Organization’s *Service availability and readiness assessment* tool.^14^ The tool focused on areas of anticipated gaps and health ministry priorities, based on previous performance indicator reports from key national hospitals. It included a total of 110 items within nine domains. In four domains (antenatal care facility assessment; emergency obstetric care services; caesarean section care; and case management of postpartum haemorrhage and eclampsia) researchers checked for the presence or absence of specific items in the medical records (e.g. medication prescriptions, nursing care plans, progress notes and discharge notes). In the remaining five domains (basic infrastructure; paediatric care; laboratory services; accessibility of guidelines and auditing efforts; and infection protection and patient safety), researchers completed the instrument using data from direct observations (e.g. for the presence or absence of infrastructure elements, and services and tools in emergency obstetric wards).

Data were collected by staff from the medical services directorate of the Ethiopian Federal Ministry of Health and regional health bureaus, the Clinton Health Access Initiative (Addis Abba, Ethiopia) and Yale University (New Haven, United States of America). The data collectors were trained to conduct observations and medical record reviews by trainers selected by the health ministry. The training was conducted in Addis Ababa over a period of 1 week.

Two or three data collectors made a 3-day site visit to each lead hospital. At visits, one researcher focused on the review of medical records (requiring about 1.5 days per hospital), while at least two other researchers conducted observations, which lasted 1.5 days, for a total sample of 24 days of observation in the 18 hospitals. In each hospital, 19 medical records were chosen using systematic random sampling from the last 12 months of birth records for a total sample of 342 records.

Baseline data were collected in June and July 2015 and follow-up data were collected in February and March 2016. We calculated overall quality scores (items met divided by total items in the instrument) as well as domain-specific scores for the different subgroups of services, at baseline and follow-up within each domain. We computed paired *t*-tests to determine the statistical significance of changes in the overall and domain-specific mean quality scores.

Between baseline and follow-up, the Ethiopia Alliance for Hospital Quality provided training on labour and delivery services for staff at the lead hospitals. Most of the training took place on site and included: refresher training on comprehensive emergency obstetric care for physicians and midwives; maternity services auditing (a new tool developed by the central team); management, analysis and use of data by quality improvements teams, hospital senior management and department heads; medical equipment management for biomedical staff; and customer service training for staff from all disciplines. The trainers were experts selected from the health ministry, Yale University and other partners and government agencies. The average training period was 3 days.

## Relevant changes

At baseline the overall mean quality score across the 18 hospitals was 65.6 (SD: 10.5) out of a possible 110 **(**Table 1). We found a significant improvement in quality scores from baseline to follow-up in eight of the nine domains (*P* < 0.05 to < 0.001). The overall summary score increased to 91.2 (SD: 12.4) out of 110 after the training intervention (*P* **<** 0.001; Table 1).

**Table 1:** Change in scores on quality of maternal and neonatal services measures from baseline to follow-up among 18 lead hospitals in Ethiopia, 2015–2016

Domains of maternal and neonatal services	No. of items per domain	Baseline^a^			Follow-up**^a^**		*P*-value^b^
		Mean quality score (SD)	% of items met^c^		Mean quality score (SD)	% of items met^c^	
Basic infrastructure score	8	5.5 (1.1)	68.8		6.7 (1.1)	83.4	0.043
Antenatal care facility assessment	9	6.6 (1.6)	73.6		8.1 (0.7)	89.8	0.010
Emergency obstetric care	33	21.8 (4.0)	66.0		28.1 (5.3)	85.1	< 0.001
Caesarean section delivery	16	10.3 (2.2)	64.6		12.1 (2.8)	75.9	0.064
Case management of postpartum haemorrhage and eclampsia	10	6.5 (1.6)	65.4		7.8 (1.3)	77.7	0.013
Paediatric care	12	6.3 (2.7)	52.8		10.2 (2.1)	84.8	< 0.001
Laboratory service	5	3.7 (1.0)	73.4		4.4 (0.7)	88.0	0.014
Guidelines and auditing	7	3.7 (1.9)	53.1		4.9 (1.8)	69.4	0.044
Infection protection and patient safety	10	6.2 (2.1)	62.2		8.9 (1.5)	89.4	< 0.001
Total maternal and neonatal service score	110	65.6 (10.5)	59.6		91.2 (12.4)	82.9	< 0.001

SD: standard deviation.

^a^ Baseline data were collected in June and July 2015; follow-up data in February and March 2016. There were no missing data from any items, domains or hospitals. Between baseline and follow-up, staff from the Ethiopia Hospital Alliance for Quality provided hospital-based training on labour and delivery services. Domain-specific quality scores for each hospital are number of items met divided by number of items in domain. Mean quality scores for each domain are the total hospital quality scores divided by 18 to obtain mean quality scores.

^b^ *t*-tests.

^c^ Percentages of items met in the domain are mean score of hospitals divided by number of items.

Notes: Lead hospitals were those selected by the Ethiopian Federal Ministry of Health based on their high performance relative to the *Ethiopian* *Hospital reform implementation guidelines* standards in Addis Ababa and the five most populous regions of Ethiopia ( Afar, Amhara, Oromia, Tigray and the Southern Nations, Nationalities and Peoples Region).

## Lessons learnt

We found that the measurement method (direct observation and medical record reviews) was generally successful, requiring a total of 3 days and two or three trained surveyors per hospital visit (Box 1). The process produced data sensitive enough to detect changes made during less than a year of quality improvement efforts. With 110 items relevant to processes of intrapartum care and facility capacity to implement such processes, the instrument provided a feasible approach to identify gaps and opportunities for improvement. We documented statistically significant improvements in almost all domains of quality at these 18 hospitals. The findings are encouraging for future quality measurement efforts in low- and middle-income countries, although the instrument would benefit from additional testing and validation. The tool and data described here represent an approach that has been embedded in Ethiopia’s hospital performance management initiative and the Ethiopia Alliance for Hospital Quality, which may enhance the sustained focus on quality of maternal and neonatal care.

Box 1 **Summary of main lessons learnt**

- The tool and process for assessing quality of intrapartum care in hospitals in Ethiopia was feasible to implement, requiring 3-day site visits by two or three data collectors.
- The process produced data sensitive enough to detect significant changes made during less than 1 year of national quality improvement efforts by the Ethiopia Hospital Alliance for Quality.
- Informed by the World Health Organization’s service availability and readiness assessment instrument, the tool provided a feasible approach to identify gaps and opportunities for improvements in quality.

The process met several key challenges. First, due to constraints of staffing, cost and time, we were unable to measure patient outcomes and had to depend on a combination of direct observation and chart reviews to obtain data. Often charts were incomplete, which was noted; more complete medical records would facilitate more precise quality measurement efforts. Second, the process required substantial financial and time investments and we could only accomplish the site visits for 18 hospitals. However, we found that having lead hospitals demonstrate the feasibility of the instrument helped promote its wider acceptance, as other hospitals seek to achieve the national recognition given to the lead hospitals. Third, clinical observations may have overestimated the use of quality processes, given that people may alter their behaviour when they know they are being observed. Additionally, it is possible that our chart reviews may have overestimated the use of quality processes, if incomplete records were more common when quality of care was worse. Our measure was therefore interpreted as an upper limit on quality processes. Nevertheless, the methods still produced useful data on changes over time. Last, we learnt that embedding such measurement in national hospital performance management efforts can help raise the visibility of such efforts, sustain needed resources for data collection and analysis, and prompt hospitals to make changes. The linking of performance, as measured by this process, to financial rewards for top performing and most improved hospitals was reported to have motivated hospitals to understand and improve their quality data. The process also helped to highlight clear gaps on which to focus future national quality improvement efforts by the medical services directorate and health ministry.
